# Photoinhibitive Properties of α-MoO_3_ on Its Composites with TiO_2_, ZnO, BiOI, AgBr, and Cu_2_O

**DOI:** 10.3390/ma16103621

**Published:** 2023-05-09

**Authors:** Endre-Zsolt Kedves, Enikő Bárdos, Alpár Ravasz, Zsejke-Réka Tóth, Szilvia Mihálydeákpál, Zoltán Kovács, Zsolt Pap, Lucian Baia

**Affiliations:** 1 Faculty of Physics, Babeş-Bolyai University, M. Kogălniceanu 1, RO-400084 Cluj-Napoca, Romania; 2 Centre of Nanostructured Materials and Bio-Nano Interfaces, Institute for Interdisciplinary Research on Bio-Nano-Sciences, Treboniu Laurian 42, RO-400271 Cluj-Napoca, Romania; 3 Department of Applied and Environmental Chemistry, University of Szeged, Rerrich tér 1, HU-6720 Szeged, Hungary; 4 Laboratory for Advanced Materials and Applied Technologies, Institute for Research, Development and Innovation in Applied Natural Sciences, Fântânele 30, RO-400294 Cluj-Napoca, Romania

**Keywords:** semiconductors, photocatalysis, α-MoO_3_, inhibition

## Abstract

Orthorhombic molybdenum trioxide (α-MoO_3_) is well known as a photocatalyst, adsorbent, and inhibitor during methyl orange photocatalytic degradation via TiO_2_. Therefore, besides the latter, other active photocatalysts, such as AgBr, ZnO, BiOI, and Cu_2_O, were assessed via the degradation of methyl orange and phenol in the presence of α-MoO_3_ using UV-A- and visible-light irradiation. Even though α-MoO_3_ could be used as a visible-light-driven photocatalyst, our results demonstrated that its presence in the reaction medium strongly inhibits the photocatalytic activity of TiO_2_, BiOI, Cu_2_O, and ZnO, while only the activity AgBr is not affected. Therefore, α-MoO_3_ might be an effective and stable inhibitor for photocatalytic processes to evaluate the newly explored photocatalysts. Quenching the photocatalytic reactions can offer information about the reaction mechanism. Moreover, the absence of photocatalytic inhibition suggests that besides photocatalytic processes, parallel reactions take place.

## 1. Introduction

Many types of semiconductors have been investigated as photocatalysts; however, combining them may be an effective approach to enhance the photocatalytic degradation yield. The photocatalyst can be activated by electromagnetic irradiation if the photon energy is higher or equal to the band gap of the exposed semiconductor. Due to excitation, charge separation occurs, resulting in active radicals in an aqueous medium, such as ·OH, ·O_2_^−^. Numerous semiconductors (TiO_2_ [1], WO_3_ [2], BiOI [3], AgBr [4], ZnO [5], Cu_2_O [6]) with various structural properties were recognized as individual photocatalysts (depending on the irradiation type: UV, visible, or NIR light) [7]. However, further efficiency improvement can be achieved by making heterostructures where the lifetime of the photogenerated charge carriers is extended. Coupling the appropriate semiconductors can induce electron and hole sinks hindering the recombination process [8,9]. Moreover, an overall band gap reduction can also be achieved so the light absorbance range of the photocatalyst will be extended in the visible light range [10].

MoO_3_ is attracting great interest because of its small band gap energy (2.7 eV), which offers visible-light-driven photocatalytic activity [11]. It was also studied in many composites to extend the band gap value or to enhance the photocatalytic performance of the base catalyst. Such an example is the well-known MoO_3_-TiO_2_ composite (47 wt.% MoO_3_), which shows better photocatalytic conversion (based on literature data) than pristine TiO_2_ or MoO_3_ [12]. ZnO, another well-known photocatalyst, presented photocatalytic enhancement under visible-light irradiation due to the presence of MoO_3_ [13]. MoO_3_ itself was also decorated with AgBr quantum dots to achieve an ultrafast dye-sensitized-assisted electron transfer process, which resulted in excellent photocatalytic activity towards rhodamine B [14]. Interestingly, papers that deal with the composites of MoO_3_ with BiOI (or any other BiOX) or Cu_2_O are scarce.

Overall, MoO_3_ is presented as a co-catalyst in photocatalytic investigations or as a visible light active photocatalyst. The actual functioning mechanism as a photocatalyst of MoO_3_ is still a debate, as it can also be an adsorbent [11,15]. Various structural characteristics (primary crystallite size, specific surface area, lattice defects, grafted functional groups, etc.) can influence whether MoO_3_ exhibits stronger adsorptive proprieties or catalytic activity. Ultimately, the interaction in suspension with the other semiconductor components in the composite could hinder or enhance photocatalytic efficiency. In our paper, we demonstrated that besides being a prominent cationic dye, adsorbents strongly inhibit the photocatalytic activity of TiO_2_ (P25). It was demonstrated that the adsorption of cationic dyes depends on the crystallographic plane ratio of α-MoO_3_ [11]. α-MoO_3_ is slightly soluble in water (pKa1 = 3.61–4.0 and pKa2 = 3.89–4.37 [16]). Hence different molybdate anions might be generated in aqueous mediums. It was suggested that dissolved MoO_4_^2−^ anions might be the cause of the photocatalytic activity decrease. It is known that the presence of anions (such as HPO_4_^2−^ CH_3_COO^−^) can inhibit photocatalytic reactions [17]. Therefore, we were interested if α-MoO_3_ can act as an inhibitor in photocatalytic processes besides other photocatalysts as well.

To demonstrate this feature, α-MoO_3_ was mixed with various active photocatalysts (such as AgBr, TiO_2_, ZnO, BiOI, and Cu_2_O). Their photocatalytic activity was assessed using two distinct organic pollutants in visible and UV irradiation. The present work aims to draw the attention of the photocatalysis community to be critical in terms of reproducibility and applicability when working with composite photocatalysts.

## 2. Materials and Methods

### 2.1. Synthesis of α-MoO_3_ and Composites

α-MoO_3_ fibers were synthesized via hydrothermal crystallization. A total of 1 g of Ammonium heptamolybdate tetrahydrate (AHM) (99.5%, NH_4_)_6_Mo_7_O_24_∙4H_2_O, Molar Chemicals, Halásztelek, Hungary) was dissolved in 90 mL of 1 M HNO_3_ (65%, Molar Chemicals, Halásztelek, Hungary), and stirred for 15 min. The transparent solution was poured into a Teflon^®^-lined stainless-steel autoclave (Toption Instrument CO., LTD., Xi’an, China) and heated up to 180 °C for 20 h. After hydrothermal crystallization, the obtained precipitate was washed with distilled water several times and dried at 40 °C for 24 h. All photocatalysts were synthesized in accordance with our previous works (TiO_2_ [18], ZnO [5], BiOI [19], AgBr [4], and Cu_2_O [6]).

The composites were mixed mechanically in an agate mortar at a ratio of 90:10 (wt.%) photocatalyst: α-MoO_3_. During the MO degradation, it was observed that 10% and 20% of α-MoO_3_ almost totally inactivated the TiO_2_, and 4% of α-MoO_3_ halved TiO_2_ photocatalytic conversion (Figure S1). As titania is one of the best photocatalysts, it means that MoO_3_ will probably affect other semiconductors in the same way. Thus, all composites comprised 10% α-MoO_3_ and 90% photocatalyst. This concentration value was chosen to ensure a difference between the pristine and MoO_3_-containing material in photoactivity.

### 2.2. Characterization Methods

X-ray diffraction (XRD) measurements were carried out with a Rigaku Miniflex II diffractometer (Cu Kα λ = 0.15406 nm, 40 kV, 30 mA, scan step size 0.02°, 20–80 (2θ°), Rigaku, Neu-Isenburg, Germany). The primary crystallite size of the identified crystal phases was estimated using the Scherrer equation.

The morphology of the composites and α-MoO_3_ was investigated with scanning electron microscopy (SEM) using a Hitachi S-4700 device (Hitachi, Tokyo, Japan). During SEM measurements, the electron beam was produced using a cold-field emission gun applying 10 kV acceleration voltage. The samples were fixed on an aluminum sample holder using conductive carbon tape.

The diffuse reflectance spectra of the samples were recorded with a JASCO-V650 spectrophotometer (JASCO, Vienna, Austria) with an integration sphere (ILV-724) between 250 and 800 nm; as a reference, BaSO_4_ was used. The band gap of the samples was calculated via Kubelka–Munk and the first-derivative method from their reflectance spectra [20].

### 2.3. The Assessment of the Photocatalytic Activity

The photocatalytic efficiency of the pristine and composite samples was evaluated by the decomposition of phenol (C_0,phenol_ = 0.5 mM) and methyl orange (MO, C_0,MO_ = 0.5 mM, C_14_H_14_N_3_NaO_3_S, 85%, NORDIC, Romania) in aqueous solutions under UV (Vilber-Lourmat T-6L UV-A, 6 × 6 W fluorescent lamps, λ_max_ ≈ 365 nm) and visible-light irradiation (6 × 6 W fluorescent lamps, λ > 400 nm) (Düwi 25920/R7S-24W), the irradiation time was 2 h. For a typical experiment, 100 mL of the model compound solution was prepared, to which the catalyst was added to set the concentration to 1 g × L^−1^. This was followed by the sonication of the mixture in the dark for 20 min to reach adsorption–desorption equilibrium. During the photocatalytic experiments, the temperature was kept at 25 °C, the homogeneity was assured by constant magnetic stirring at 400 rpm, and the oxygen concentration was maintained by providing constant air supply (30 L × h^−1^) during the measurements. Samples were taken each 10 min in the first hour and 20 min in the second hour.

The phenol concentration was monitored by high-performance liquid chromatography (HPLC) with a device consisting of a Merck Hitachi L-7100 low-pressure gradient pump and a Merck-Hitachi L-4250 UV–Vis detector (λ_detection_ = 210 nm) (HPLC, L-7100, Merck-Hitachi, Darmstadt, Germany), using a 50%–50% methanol/water mixture as the eluent. The MO concentration was monitored using the Jasco UV-Vis spectrophotometer (λ_detection_ = 464 nm). It should be mentioned that the pristine α-MoO_3_ was inactive in all the photocatalytic experiments. Moreover, no photolysis was detected for MO and phenol under UV or under visible light.

## 3. Results and Discussion

**Figure 1:**
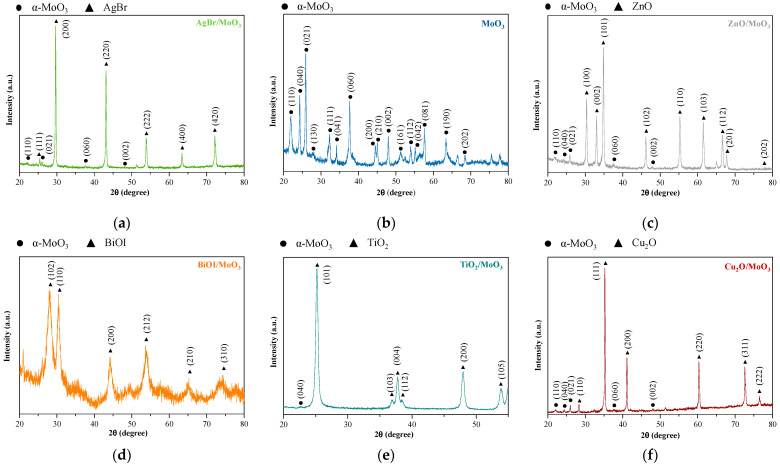
X-ray diffractograms of the as-prepared composites: (**a**) AgBr/MoO_3_, (**b**) α-MoO_3_, (**c**) ZnO/MoO_3_, (**d**) BiOI/MoO_3_, (**e**) TiO_2_/MoO_3_, and (**f**) Cu_2_O/MoO_3_ composites’ XRD patterns.

**Figure 2:**
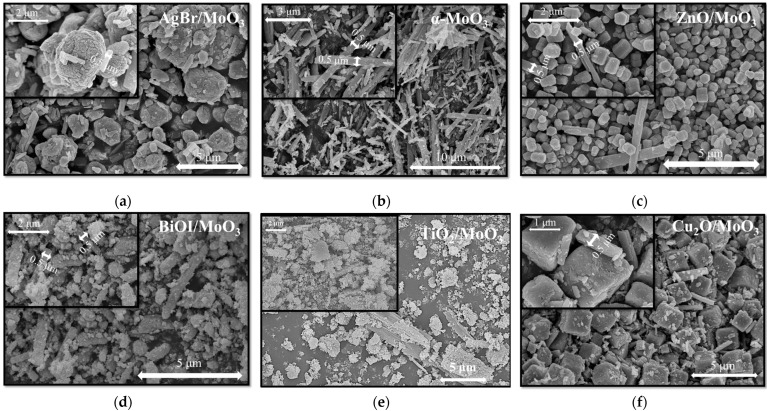
Micrographs of the composites: (**a**) AgBr/MoO_3_, (**b**) α-MoO_3_, (**c**) ZnO/MoO_3_, (**d**) BiOI/MoO_3_, (**e**) TiO_2_/MoO_3_, and (**f**) Cu_2_O/MoO_3_.

The as-prepared semiconductors and their composites were analyzed via XRD and SEM measurements to ascertain the reproducibility of photocatalysts and the presence of MoO_3_ in the composites. The structure of the as-prepared pristine photocatalysts was reproducible: TiO_2_ was obtained in the form of anatase [18], BiOI in tetragonal matlockite [19], ZnO in hexagonal wurtzite [5], AgBr [4], and Cu_2_O in cubic form [6] (Figure 1). The as-prepared MoO_3_ presented an orthorhombic phase with (021) a dominant crystallographic plane (Figure 1b). Despite the mechanical mixing, the reflections of α-MoO_3_ were faintly visible on the XRD patterns of the composites (it was the case of AgBr/MoO_3_, Cu_2_O/MoO_3_, and ZnO/MoO_3_, while in the case of BiOI/MoO_3_, no MoO_3_ reflections were noticed). However, the SEM micrographs showed that the characteristic α-MoO_3_ fibers were present in all of the composites (Figure 2). Based on these two structural characteristics, it was considered that the active photocatalysts were successfully reproduced and the fibrous α-MoO_3_ was present in these composites (the synthesis of α-MoO_3_ was carried out in such a way that the MoO_3_ particles will be crystalline—that is why 20 h was considered as the minimal crystallization time, which influences the quality of the final product of course [21]). Using the Scherrer equation, the crystallite sizes of the samples were estimated. Unfortunately, in the composites, the peaks corresponding to the MoO_3_ phase were not suitable for the calculation, but the other photocatalysts showed strong, intensive peaks. The crystallite size of the other component semiconductors showed no significant difference compared to the pristine phase. Hence the calculation results are as follows; TiO_2_—14.2 nm, AgBr—36.8 nm, ZnO—38.0 nm, and BiOI—14.3 nm, while for Cu_2_O, the calculation was not possible due to its microcrystalline structure. This indicates that the mechanical mixing was successful and induced no deviation in the crystal structure.

The SEM micrographs (Figure 2) also revealed the size and arrangement of the crystals. In each of the composite samples, the diameter of the MoO_3_ rods was preserved (~0.5 µm, Figure 2b shows the pristine MoO_3_). The AgBr particles appeared (Figure 2a) as hierarchical structures with variable sizes (from 1 up to 5 µm), while the shape of the particles was random. In the case of the ZnO-base composite, the MoO_3_ particles were larger than ZnO (which were smaller than 400 nm, Figure 2c). In the case of BiOI and TiO_2_, the particles were randomly aggregated and covered, in most cases, the available MoO_3_ nanorods (Figure 2d,e). No clear size evaluation or particle size distribution can be carried out. The situation is quite different in the case of Cu_2_O. The crystals were cubes (1–2.5 µm), which were alongside the MoO_3_ rods (Figure 2f).

**Figure 3:**
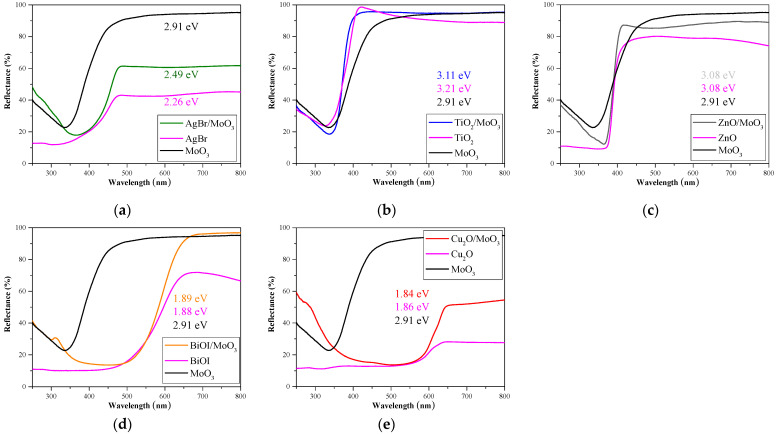
DRS spectra and their respective band gap values: (**a**) AgBr/MoO_3_, (**b**) TiO_2_/MoO_3_, (**c**) ZnO/MoO_3_, (**d**) BiOI/MoO_3_, and (**e**) Cu_2_O/MoO_3_.

Based on the measured optical properties (Figure 3) and our previous work, TiO_2_ and ZnO should exhibit photocatalytic activity in the UV range and Cu_2_O, AgBr, and BiOI in the visible range. The calculated band gap value for α-MoO_3_ (2.91 eV) suggested that it is a promising visible-light active photocatalyst. In composites, the presence of α-MoO_3_ did not or only slightly affected the band gap values of the pristine photocatalysts (Figure 3). The band gap value of TiO_2_ was shifted by 0.1 eV toward the visible range, while in the case of ZnO, it did not change at all. The other three photocatalysts presented an increased reflectance in the visible region; however, the calculated band gap for AgBr increased by 0.23 eV, and for BiOI and Cu_2_O, the change was irrelevantly small (<0.05 eV) (Figure 3). The first derivative method is more advised to determine the band-gap energies of composite materials because it does not generalize the spectral values and could reveal the band-gap values of the constituent phases. However, in the present case, the values showed no significant difference compared to the Kubelka–Munk method, and the peak corresponding to MoO_3_ was not observable in the derivative spectra. This could also be explained by the small percentage of the MoO_3_ phase in the composites.

Photocatalysis is based on the utilization of charge carriers: the organic molecule can be oxidized, whether directly via holes, or by different types of radicals (⋅O_2_, ·OH). Depending on the MO concentration its photocatalytic decomposition is a very common example of both hole (>1.6 × 10^−4^ M) and hydroxyl oxidation (<1.6 × 10^−4^ M) [22], while phenol degradation is driven by hydroxyl radicals to form intermediate hydroxylated compounds—such as catechol, benzoquinone, and other compounds [23].

**Figure 4:**
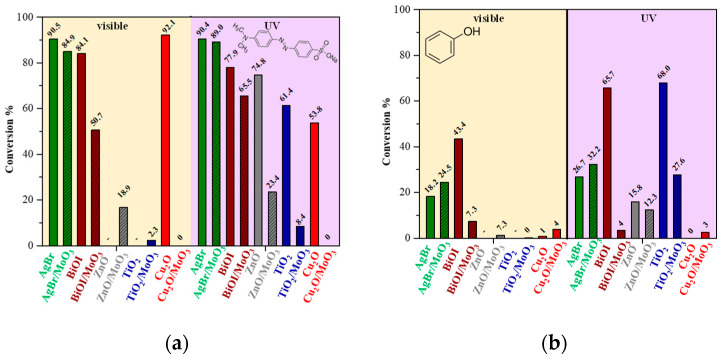
The photocatalytic degradation of (**a**) methyl orange and (**b**) phenol via the pristine photocatalysts and the as-prepared α-MoO_3_-containing composites in UV and visible irradiation.

The photocatalytic efficiency values are presented in Figure 4. Pristine photocatalysts reached higher activity in the degradation of methyl orange compared to phenol, except in the case of TiO_2_ (Figure 4). Higher photocatalytic conversion for MO could be achieved because its degradation mechanism consists of both radicals (hydroxyl and superoxide radicals) and photogenerated holes. Anatase TiO_2_ and ZnO were active only in UV due to their optical properties. Cu_2_O presented outstanding activity in the degradation of MO in visible light (92% conversion), while in UV, adsorption was predominant (Figure S2). Cu_2_O was not active in the photodegradation or adsorption of phenol. The literature also presents that Cu_2_O is both a visible-light-active photocatalyst and an adsorbent for MO [24,25]. BiOI and AgBr, independently from the light source, presented stable activity in MO decomposition. However, phenol degradation reached higher conversion under UV illumination (Figure 4).

In the present work, the motivation was to assess the effect of α-MoO_3_ upon the above-presented pristine photocatalysts and their activity. We demonstrated before that α-MoO_3_ reduces the photocatalytic activity of TiO_2_, although band gap narrowing or the electron–hole recombination suppression might occur [1]. Our current results confirm that not only TiO_2_ activity but the activity of photocatalysts (ZnO, BiOI, AgBr, and Cu_2_O) can be hindered in the presence of α-MoO_3_. As it was mentioned before, inhibition might occur due to the formation of HMoO_4_^−^ and MoO_4_^2−^ anions, and those may function as hole scavengers, such as HPO_4_^2−^ and CH_3_COO^−^ [16]. The presence of MoO_4_^2−^ anions should induce a pH drop. However, an acidic medium does not affect the degradation rate of MO via TiO_2_. Its apparent rate constant is unchanged between pH 3 and 8 [17].

The mentioned Mo-species (HMoO_4_^−^ and MoO_4_^2−^ anions) may be pretty challenging to follow under the current circumstances as the equilibrium constant (or the pKa values: pKa_1_ = 3.61–4.0 and pKa_2_ = 3.89–4.37, [11]) for the hydrolysis of MoO_3_ suggests that the solubilization is not the favored reaction. This means that a smaller (nM or a few µM) concentration of Mo species can always be found in the solution. However, if hydrolysis is the dominant process, MoO_3_ would be dissolved entirely. Furthermore, during the hole-scavenging process, after the electron transfer, MoO_3_ may be re-deposited. Hence the presence of HMoO_4_^−^ and MoO_4_^2−^ is transitory and challenging to follow. Moreover, the activity decrease can occur due to the OH radical scavenging effect of MoO_3_ [26].

Light shielding via MoO_3_ might be another reason for inactivity. However, this scenario is less likely, because the amount of MoO_3_ used in the experiment was 10 wt.%, which is not much, considering that in the literature, sometimes nearly 50 wt.% of MoO_3_ (33 molar%) was used. Still, photoactivity was registered for these samples [12]. Further, when the influence of the MoO_3_ content was investigated on titania photocatalysts, it was found that the activity decrease was not linear with the MoO_3_ content increase [11]. Hence, the presence of α-MoO_3_ or its anions should be the cause that inactivates the photocatalysts.

In MO photodegradation, all the composite materials presented lower conversion than without α-MoO_3_ (Figure 4a, Figure S2). TiO_2_ and ZnO presented a ~50% conversion drop, which is particularly high as only 10% photocatalyst was substituted with α-MoO_3_. The photocatalytic activity of Cu_2_O completely disappeared, and the tendency to adsorb MO has ceased. Finally, BiOI conversion was least affected by α-MoO_3_; only 15–30% conversion drop was observed. The inhibition varied depending on the form of illumination. A more intense inhibition was observed during visible-light irradiation. On the contrary, the activity of AgBr in MO decomposition was hindered only in UV, though after 2 h illumination, the AgBr/MoO_3_ composites reached a similar conversion to pristine AgBr. An opposite behavior was observed when ZnO was analyzed. Under visible-light irradiation, the composite achieved ~10% conversion, while the bare ZnO was inactive. This suggests that, to some extent, MoO_3_ can be activated as well.

Phenol photocatalytic decomposition was also inhibited by α-MoO_3_ as the methyl orange decomposition (Figure 4b, Figure S3). ZnO and Cu_2_O photocatalytic activity or adsorption were negligible during phenol degradation. Hence their composite activity was not counted. TiO_2_ presented a ~40% conversion drop, similar to MO degradation. In the case of BiOI, the inhibition was higher (40–60% conversion drop) compared to MO photocatalytic tests. In both irradiation cases, BiOI photocatalytic activity was decreased in the presence of α-MoO_3_. The pollutant conversion was below ~10%, although, without MoO_3_, BiOI phenol decomposition reached 65.7% in UV and 43.4% in vis. Since all the other photocatalysts were inhibited by α-MoO_3_ regardless of the model pollutant or irradiation, as a surprising fact, phenol decomposition by AgBr slightly increased under UV or visible irradiation. Compared to the other photocatalysts, AgBr is known to be unstable during photocatalytic treatment. AgBrO_3_, Ag, and Ag_2_O can be formed on AgBr surfaces during the photocatalytic process [4]. The formation of these species might be the first cause of the MO and phenol degradation and not solely the photocatalysis, which can be hindered by α-MoO_3_. It should be noted that in the case of ZnO-based composites, a small amount of phenol degradation occurs under visible-light irradiation (~7%), emphasizing the issue raised for this composite in the case of MO degradation.

Light screening could be another issue for catalyst inactivation. However, this was not the case. The phenomenon requires a material that absorbs light intensively instead of the main catalyst. The simultaneous prerequisite conditions are a low band-gap energy, a significant difference (above 1.0 eV) between the band-gap values, and low (below 30%) reflectivity of the minor composite component (MoO_3_). Neither of these conditions is valid at the same time.

## 4. Conclusions

Our results suggested that α-MoO_3_, next to an active photocatalyst, predominantly acts as a photocatalytic inhibitor rather than an enhancement factor, as stated frequently in the literature. α-MoO_3_ inhibition was manifested by a decrease in the activity of the photocatalysts, whether MO or phenol was decomposed under visible or UV light. Only the activity of AgBr was unaffected, yet it is known that AgBr is unstable during photocatalysis. Therefore, not only photocatalysis occurs, but the formation of Ag compounds, which might oxidize the organic pollutants, and that cannot be inhibited by α-MoO_3_. We deduced that α-MoO_3_, or its anions, can inhibit photocatalytic processes, and its inhibition efficiency varies depending on the type of irradiation, the pollutant, and the photocatalyst. Moreover, these results point out that the current scientific community does not present those very important results in which a negative aspect is presented regarding a specific material. It should be noted that charge transfer inhibition and inactivation may influence other research areas besides photocatalysis (e.g., supercapacitive materials, sensor applications, energy storage devices). Hence appropriate documentation of negative phenomena should be considered. As α-MoO_3_ tends to transform in aqueous media, environmental aspects should also be considered, as Mo is a heavy metal, after all.

## Data Availability

The data presented in this study are available on request from the corresponding author. The data are not publicly available due to their implication in future industrial research.

## Supplementary Materials

The following supporting information can be downloaded at: https://www.mdpi.com/article/10.3390/ma16103621/s1, Figure S1. Photocatalytic degradation of MO with TiO_2_/MoO_3_ composites in different weight percentages under UV irradiation: (a) TiO_2_, (b) 96/4, (c) 90/10, and (d) 80/20; Figure S2. Methyl orange degradation curves under UV and visible irradiation: (a) AgBr, AgBr/MoO_3_, (b) TiO_2_, TiO_2_/MoO_3_, (c) ZnO, ZnO/MoO_3_, (d) BiOI, BiOI/MoO_3_, and (e) Cu_2_O, Cu_2_O/MoO_3_; Figure S3. Phenol degradation curves under UV and visible irradiation: (a) AgBr, AgBr/MoO_3_, (b) TiO_2_, TiO_2_/MoO_3_, (c) ZnO, ZnO/MoO_3_, (d) BiOI, BiOI/MoO_3_, and (e) Cu_2_O, Cu_2_O/MoO_3_.
