# Supplementary material for: Photoinhibitive Properties of α-MoO3 on Its Composites with TiO2, ZnO, BiOI, AgBr, and Cu2O
Source: Materials (Basel). 2023 May 9;16(10):3621. doi: 10.3390/ma16103621 (PMC10223306; doi:10.3390/ma16103621)
Supplement: Supplementary file 1 [file materials-16-03621-s001.zip › materials-2193478-supplementary.pdf]

materials

Supplementary Materials

Photoinhibitive Properties of α-MoO3 on its Composites with TiO2, ZnO, BiOI, AgBr, and Cu2O

Endre-Zsolt Kedves [1,2,3], Enikő Bárdos [3], Alpár Ravasz [2], Zsejke-Réka Tóth [2,3], Szilvia Mihálydeákpál [3], Zoltán Kovács [1,3], Zsolt Pap [2,3,4,*] and Lucian Baia [1,2,4,*]

1 Faculty of Physics, Babeş-Bolyai University, M. Kogălniceanu 1, RO-400084 Cluj-Napoca, Romania
2 Centre of Nanostructured Materials and Bio-Nano Interfaces, Institute for Interdisciplinary Research on Bio-Nano-Sciences, Treboniu Laurian 42, RO-400271 Cluj-Napoca, Romania
3 Department of Applied and Environmental Chemistry, University of Szeged, Rerrich tér 1, HU-6720 Szeged, Hungary
4 Laboratory for Advanced Materials and Applied Technologies, Institute for Research, Development and Innovation in Applied Natural Sciences, Fântânele 30, RO-400294 Cluj-Napoca, Romania
* Correspondence: pzsolt@chem.u-szeged.hu (Z.P.); lucian.baia@ubbcluj.ro (L.B.)

Citation: Kedves, E.-Z.; Bárdos, E.; Ravasz, A.; Tóth, Z.-R.; Mihálydeákpál, S.; Kovács, Z.; Pap, Z.; Baia, L. Photoinhibitive Properties of α-MoO_3 on Its Composites with TiO_2, ZnO, BiOI, AgBr, and Cu_2O. *Materials* **2023**, *16*, 3621. https://doi.org/10.3390/ma16103621

Academic Editor: Roberta G. Toro

Received: 10 February 2023
Revised: 13 April 2023
Accepted: 20 April 2023
Published: 9 May 2023

Copyright: © 2023 by the authors. Licensee MDPI, Basel, Switzerland. This article is an open access article distributed under the terms and conditions of the Creative Commons Attribution (CC BY) license (https://creativecommons.org/licenses/by/4.0/).

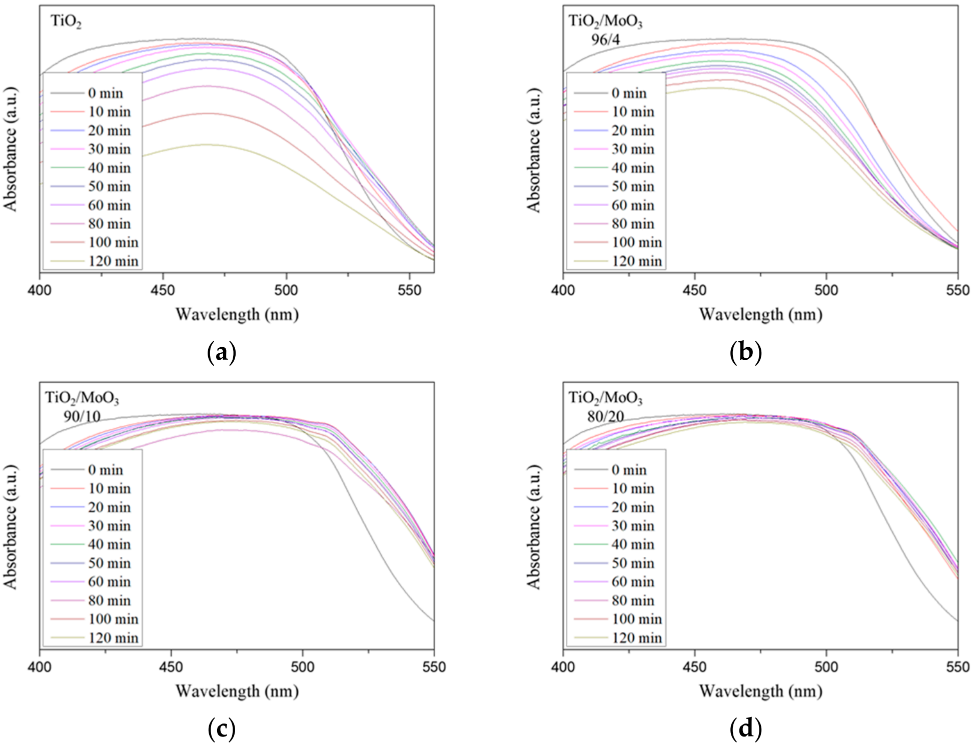

Figure S1. Photocatalytic degradation of MO with TiO_2/MoO_3 composites in different weight percentages under UV irradiation: (**a**) TiO_2, (**b**) 96/4, (**c**) 90/10, and (**d**) 80/20.

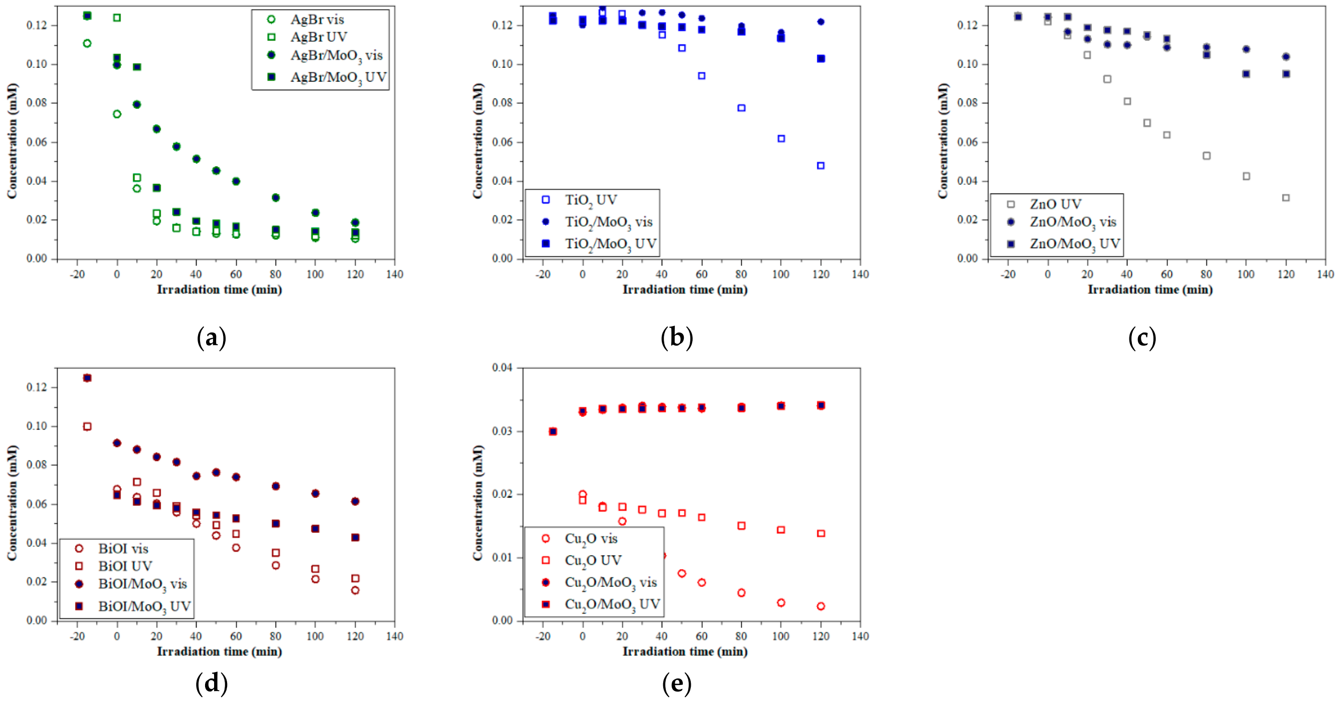

Figure S2. Methyl orange degradation curves under UV and visible irradiation: (**a**) AgBr, $AgBr/MoO_3$, (**b**) TiO_2, TiO_2/MoO_3, (**c**) ZnO, ZnO/MoO_3, (**d**) BiOI, $BiOI/MoO_3$, and (**e**) Cu_2O, Cu_2O/MoO_3.

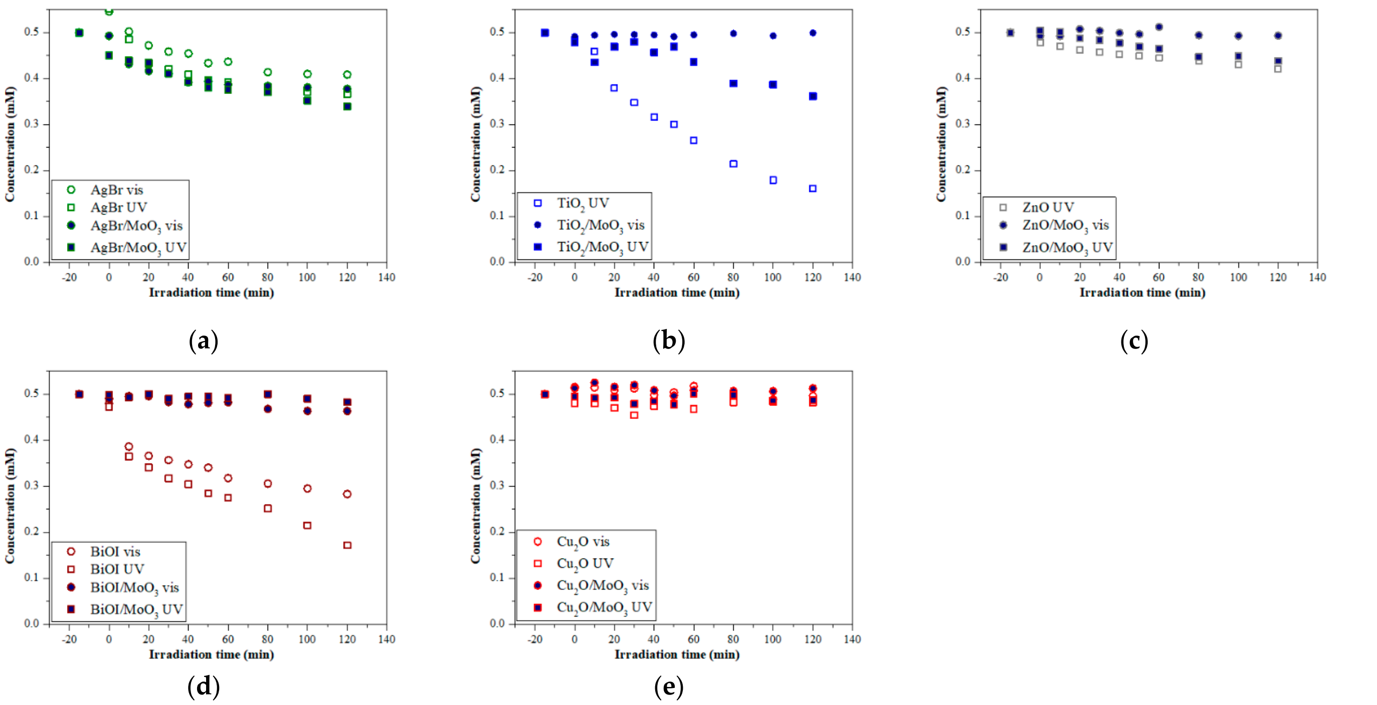

Figure S3. Phenol degradation curves under UV and visible irradiation: (**a**) AgBr, AgBr/MoO_3, (**b**) TiO_2, TiO_2/MoO_3, (**c**) ZnO, ZnO/MoO_3, (**d**) BiOI, BiOI/MoO_3, and (**e**) Cu_2O, Cu_2O/MoO_3.

Disclaimer/Publisher's Note: The statements, opinions and data contained in all publications are solely those of the individual author(s) and contributor(s) and not of MDPI and/or the editor(s). MDPI and/or the editor(s) disclaim responsibility for any injury to people or property resulting from any ideas, methods, instructions or products referred to in the content.
